# Enzyme-Based Listericidal Nanocomposites

**DOI:** 10.1038/srep01584

**Published:** 2013-04-02

**Authors:** Kusum Solanki, Navdeep Grover, Patrick Downs, Elena E. Paskaleva, Krunal K. Mehta, Lillian Lee, Linda S. Schadler, Ravi S. Kane, Jonathan S. Dordick

**Affiliations:** 1 Department of Chemical and Biological Engineering, Center for Biotechnology & Interdisciplinary Studies, and Rensselaer Nanotechnology Center, Rensselaer Polytechnic Institute, Troy, NY 12180, USA; 2 Department of Materials Science and Engineering, Center for Biotechnology & Interdisciplinary Studies, and Rensselaer Nanotechnology Center, Rensselaer Polytechnic Institute, Troy, NY 12180, USA; 3 These authors contributed equally to this work.

## Abstract

Cell lytic enzymes represent an alternative to chemical decontamination or use of antibiotics to kill pathogenic bacteria, such as listeria. A number of phage cell lytic enzymes against listeria have been isolated and possess listericidal activity; however, there has been no attempt to incorporate these enzymes onto surfaces. We report three facile routes for the surface incorporation of the listeria bacteriophage endolysin Ply500: covalent attachment onto FDA approved silica nanoparticles (SNPs), incorporation of SNP-Ply500 conjugates into a thin poly(hydroxyethyl methacrylate) film; and affinity binding to edible crosslinked starch nanoparticles *via* construction of a maltose binding protein fusion. These Ply500 formulations were effective in killing *L. innocua* (a reduced pathogenic surrogate) at challenges up to 10^5^ CFU/ml both in non-growth sustaining PBS as well as under growth conditions on lettuce. This strategy represents a new route toward achieving highly selective and efficient pathogen decontamination and prevention in public infrastructure.

With an estimated 2000 hospitalizations and 500 deaths annually in the United States alone, listeriosis is the leading cause of death among diseases caused by foodborne bacterial pathogens^1^. *Listeria monocytogenes* is a foodborne pathogen associated with severe infections and high mortality^2^,^3^,^4^, and is one of most prevalent pathogens found in the food supply^5^. Although treatment of listeriosis is feasible with a course of antibiotics^6^,^7^, increased resistance of pathogenic listeria strains remains a public health challenge^8^,^9^,^10^. Therefore, it is critical to reduce listeria contamination both in the food infrastructure and the food itself, prior to ingestion by the consumer. Various chemical and physical decontamination strategies are in use today, including those based on the use of hydrogen peroxide, quaternary ammonium compounds, bleach, sodium or potassium lactate^8^,^11^,^12^, hot water washes, among others. These methods cannot be used on foodstuffs directly, and the corrosiveness of most of the chemical decontaminants limit their use and effectiveness in the food infrastructure. There remains, therefore, an acute need in the food industry to develop an effective and non-toxic approach to eliminate food-borne pathogens that impact equipment/infrastructure, e.g., thaw and cold rooms, conveyor belts, grinders, commercial kitchens, and packaging materials. Surfaces for application include steel and rubber (meat conveyor belts), ceramic/tile (cold/storage rooms), and aluminum, concrete/stone, sheetrock (countertops).

In the past two decades, cell lytic enzymes isolated from bacteriophages have emerged as unique antimicrobial agents that possess exceptionally high specificity and an ability to act against multi-drug resistant microbes^13^,^14^,^15^. Ply500 is a characterized listeria cell lytic enzyme^16^, with peptidase activity against *L. monocytogenes* serotypes 4 (including 4b^16^,^17^ that is responsible for more than 50% of the reported cases of listeriosis^18^), 5, and 6. As with many known endolysins, Ply500 consists of two domains; the N-terminal enzymatic active domain (EAD), which cleaves the bacterial cell wall, and the C-terminal cell binding domain (CBD), which targets the enzyme specifically to its substrate in the bacterial cell wall envelope^16^,^17^. Due to their chimeric nature, attachment of active endolysins onto various surfaces is complicated by the need for both the N- and C-terminal domains to access listeria's wall (peptidoglycan), which likely requires a compromise for proper orientation. Herein, we take advantage of the intrinsic stabilization of enzymes on nanomaterials^19^,^20^,^21^ and prepare Ply500-nanoparticle conjugates that can be used in stabilized catalyst formulations and can be embedded within a simple polymeric film, thereby generating a safe surface for removal of listeria and enhancing food safety.

## Results

**Figure 1:**
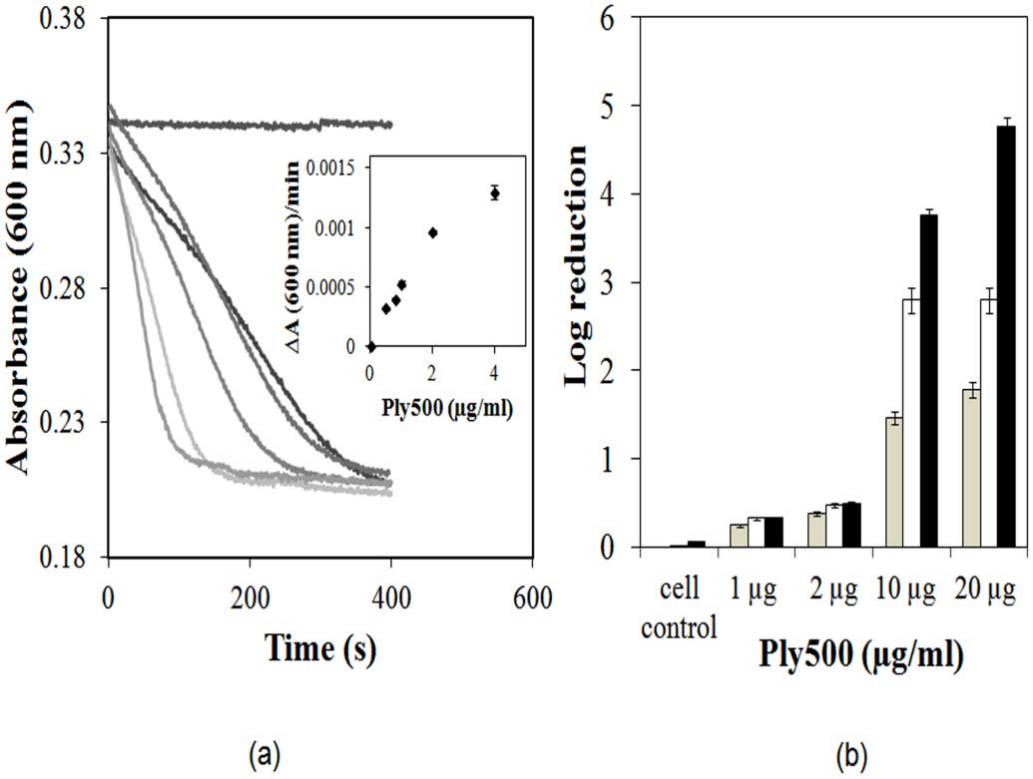
(a) Activity of Ply500 against isolated *L. innocua* cell walls as measured by decrease in turbidity at 600 nm in Tris-HCl buffer (50 mM, pH 8.0) containing 250 mM NaCl. Curves (top to bottom) represent the no-enzyme cell wall control, 0.5, 0.8, 1, 2 and 4 μg/ml Ply500. All assays were performed in triplicate and the curves represent the mean value. Inset represents slopes as a function of Ply500 concentration. (b) Effect of Ply500 concentration on listeria killing using a viable plate counting assay after 3 h (grey), 6 h (white) and 24 h (black). *L. innocua* was grown in BHI media for 7 h. The assay cell challenge was 10^5^ CFU/ml with a total reaction volume of 1-ml. Error bars represents standard deviations from triplicate measurements.

6x-His-Ply500 was produced in *E. coli* and purified by Ni-NTA chromatography (see details in Supplementary information). The purified protein (33.4 kDa) was stored in Phosphate Buffered Saline (PBS) at 4°C at concentrations <1 mg/ml to avoid aggregation. Unlike many phage endolysins^22^, Ply500 was active and stable as a monomer (90 ± 3% of total purified enzyme; see details in Supplementary Information and Supplementary Fig. S1). The monomer was stable for at least 7 days when stored in solution or as a lyophilized powder at 4°C, with <5% of monomer converted into dimer. The catalytic activity of Ply500 was initially assessed via breakdown of isolated cell walls of *L. innocua* (Fig. 1a), with optimum assay conditions of pH 8.0 in 250 mM NaCl and 50 mM Tris buffer, translating into a specific activity of 3000 ± 300 U/mg. The rate of turbidity decrease was essentially linear with enzyme concentration in a range of 0.5 to 2.0 μg/ml (Fig. 1a, Inset). Irrespective of the concentration of Ply500 used, the final turbidity obtained after lysis was ~60% of the initial value, indicating that the enzyme is rather specific and cannot cleave the cell wall peptidoglycan into extremely small, and more soluble fragments.

To date, Ply500 has not been employed for actual cell killing assays. Therefore, to obtain accurate cell killing activity, we proceeded to establish a protocol for the antimicrobial/plating assay of Ply500. An appropriate amount of filter-sterilized enzyme (1–20 μg) was incubated with listeria (10^5^ CFU/ml) in 1 ml of sterilized PBS buffer containing 0.1% (v/v) Tween 80 followed by plating on BHI agar plates (Fig. 1b). Ply500 was highly active against *L. innocua*; for a 10^5^ CFU/mL listeria cell challenge, a 3-log and close to 5-log reduction was obtained after 24 h using 10 and 20 μg/mL of enzyme, respectively (Fig. 1b). To ascertain whether the residual listeria cells were naturally resistant to Ply500, we performed six repeated cycles of killing, outgrowth, and killing and observed no resistance against Ply500 (see details in Supplementary Information).

**Figure 2:**
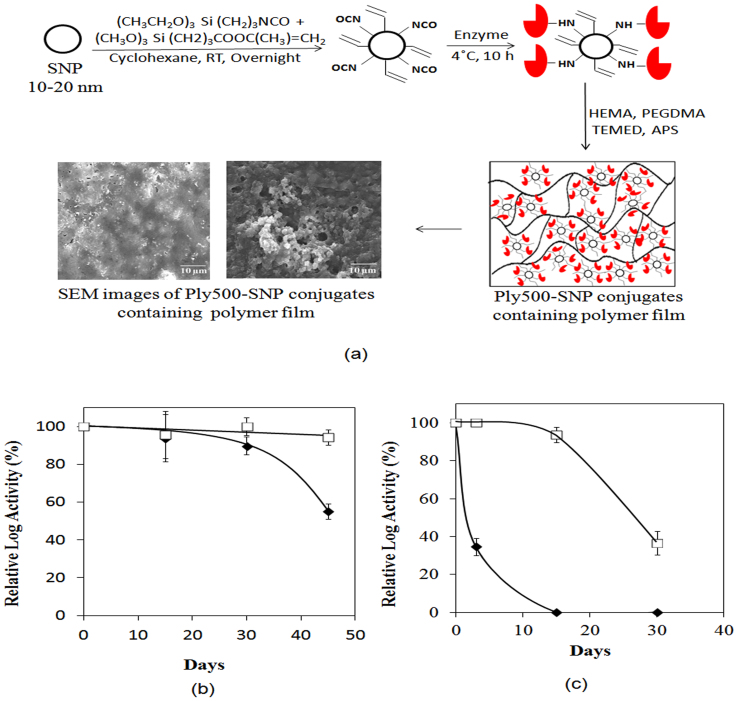
(a) Schematic representation of covalent immobilization of Ply500 on silica nanoparticles followed by co-polymerization with HEMA to form an anti-listeria polymeric film. Storage stability of native Ply500 (20 μg) (♦) and Ply500-SNP (30 μg) (□) in sterile PBS at (b) 4°C and (c) 25°C. The amount (in μg) represents the Ply500 content of the formulation. Error bars represents standard deviations from triplicate measurements.

Endolysins require both the N- and C-domains to be in proper orientation to access the substrate (bacterial cell wall) for activity^17^. Thus, even small limitations in accessibility and enzyme conformational deformation due to immobilization would likely result in very low observed activity. For these reasons, we hypothesized that immobilization onto a surface that is similar in size to the enzyme itself, yet possessing the virtues of an immobilization support, would yield highly active and stable Ply500 formulations. To that end, we chose Silica nanoparticles (SNPs) as the conjugation material. SNPs are cost effective, inert and are considered as GRAS by the U.S. Food and Drug Administration^23^, which enable them to be used in the food industry^23^. SNPs were functionalized with NCO and methacrylate groups (Fig. 2a), and the methacrylate functionalization was qualitatively confirmed through reaction of the methacrylate group double bond with Br_2_, followed by X-ray photoelectron spectroscopy (XPS) analysis of the Br signature (see details in Supplementary Information; Supplementary Fig. S2). Titration of the functionalized SNPs with benzylamine indicated that functionalization of both NCO and methacrylate groups is 0.1 ± 0.01 mmol/g SNPs (see details in supplementary information). The enzyme loading was 0.14 ± 0.03 mg Ply500/mg SNPs. Dynamic light scattering was used to determine the size of SNPs in PBS buffer (containing 0.1% (v/v) Tween 80) at each step of modification (see details in Supplementary Information; Supplementary Table S1). Modification with NCO and methacrylate SNPs resulted in formation of SNP aggregates of 65 ± 18 nm (Supplementary Table S1). Immobilization of Ply500 led to further crosslinking and increased aggregate size of 81 ± 29 nm (Supplementary Table S1).

We evaluated the effect of covalent attachment on the bactericidal activity of Ply500 (Table 1). Ply500-SNP conjugates showed ~33% retention of activity, as determined by the cell plating assay (on basis of number of listeria cells killed per mg of Ply500 in the conjugates) (Table 1). While free Ply500 (20 μg/ml) showed complete cell killing against a challenge of 10^5^ CFU/ml in 24 h, a similar bactericidal effectiveness required Ply500-SNP conjugates containing 60 μg/ml of Ply500 (Table 1), indicating some loss of enzyme activity upon immobilization. The SNP no-enzyme control was ineffective in killing listeria. The SNP-Ply500 conjugates were substantially more stable than the free enzyme at both 4 and 25°C, temperatures representative of storage and operational conditions, respectively (see details in supplementary information). At 4°C the free enzyme lost 50% of its log killing (calculated on the basis of log reduction per mg of Ply500) after 40 days, whereas the SNP-Ply500 conjugates retained >95% of its initial log killing during this time (Fig. 2b). More striking was the stabilization at 25°C, where the native enzyme was completely deactivated after 15 days, while with the nanoparticle conjugates retaining >95% log killing during this time (Fig. 2c). The increased stability of SNP-Ply500 conjugates obtained was in agreement with previous reports, which demonstrate that protein stability is enhanced by the highly curved surfaces of nanosupports^19^,^24^,^25^,^26^. The retention of bactericidal activity and high storage stability were sufficiently encouraging to proceed with incorporating these conjugates into a polymer film.

The Ply500-SNP conjugates containing methacrylate groups were co-polymerized with HEMA and polyethylene glycol dimethylacrylate (PEGDMA), as crosslinker, in the presence of ammonium persulphate (APS) and tetramethyl ethylenediamine (TEMED) as initiators (Fig. 2a). The Ply500-SNP conjugates were dispersed throughout the film, as shown via scanning electron microscopy (SEM) (Fig. 2a). The polymer film containing Ply500 conjugates was then tested for bactericidal activity against *L. innocua* by incubating the film in 2 ml of a suspension containing 10^5^ CFU/ml for 24 h (Table 1). This incubation resulted in a 1.6-log reduction in the number of *L. innocua* cells, whereas the control film with only SNPs (i.e., without Ply500) was devoid of antimicrobial activity. Next, we confirmed that the activity of the film was not due to leaching of Ply500 into the solution. Specifically, the Ply500-SNP containing film was incubated in PBS (with 0.1% (v/v) Tween 80) for 24 h, the film was removed, and the solution was tested for free protein leached using the BCA assay. No free protein was observed in the washes; however, the detection limit of the BCA assay is not sufficiently low to truly assess for leached enzyme. A more sensitive antimicrobial activity assay, therefore, was performed on the washes, which also yielded no evidence of protein leaching from the film. To ensure that Ply500-SNP conjugates were not simply leaching in an inactive form, we used a highly sensitive surrogate fluorescent protein, green fluorescent protein (GFP), incorporated into pHEMA in a manner identical to that of Ply500 (see details in Supplementary information, Supplementary Fig. S3). Specifically, GFP was immobilized onto the SNPs, with a measured loading of 0.04 mg/mg SNPs (Supplementary Fig. S3(a)). Then the polymer film was cast with the embedded GFP-SNP conjugates. The resulting film containing GFP-SNP conjugates was incubated in PBS (with 0.1% (v/v) Tween 80), respectively, for 24 h and the film was then removed from the buffer. The resulting fluorescence of the buffer solutions was no higher than that for controls (polymer films with SNP without GFP), indicating no significant protein leaching from the film (Supplementary Fig. S3 (b)). Collectively, these results indicate that the anti-listeria activity of the films was a result of Ply500 activity within the film.

Ready to eat (RTE) foods, which are stored at 4°C and are not cooked properly prior to consumption, are the main source of listeria outbreaks. Cross-contamination in RTE foods comes from adhered cells or listeria biofilms in cold storage equipment. We therefore examined adherence and growth of *L. innocua* cells on Ply500-containing polymer films. The cells were allowed to adhere and grow on the pHEMA polymer films for 48 h at the typical food storage and processing temperature of 4°C. No *L. innocua* cells were found on Ply500-SNP-containing films as compared to 50 ± 10 cells found on the control (no-enzyme) film. Hence, the SNP-Ply500-containing pHEMA films were able to prevent the viability of adhered listeria cells.

**Figure 3:**
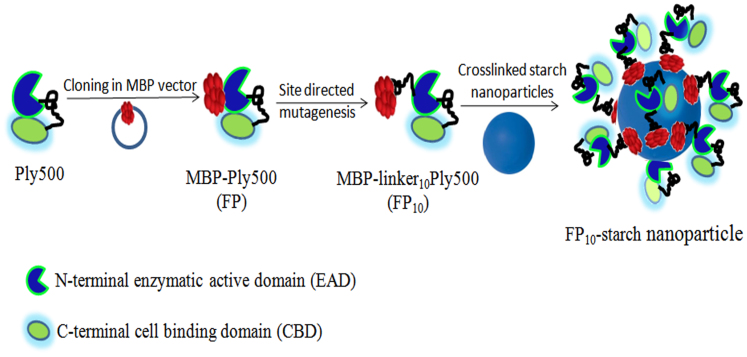
Schematic representation of oriented affinity immobilization of MBP-linker_10_ Ply500 fusion protein on crosslinked starch particles *via* MBP.

An additional challenge in the food industry is the need for safety in packaging and food transport. Indeed, nearly 40% of the food produced in the world is never eaten^27^,^28^. Therefore, modification of food packaging to prevent growth of foodborne pathogens is highly desirable. Along these lines, we examined the use of starch as an edible, cheap material, which is often sprayed into the packaging as a powder layer on meat products^29^. We took advantage of the natural affinity of a maltose binding protein (MBP) fused to Ply500 to selectively attach the enzyme to ca. 100 nm starch nanoparticles (Fig. 3).

The MBP-Ply500 (FP) fusion protein was constructed to contain an N-terminal MBP fusion and a C-terminal 6x-His Tag (Fig. 3). Following optimization of enzyme induction and expression in BL21 cells, the fusion protein was purified using Ni-NTA chromatography (see details in Supplementary Information, Supplementary Fig. S4). The purified MBP-Ply500 (FP) was then evaluated for activity against *L. innocua* using the viable plate assay, as was used for native Ply500 (Table 1). Free Ply500 (20 μg/ml) challenged with 10^5^ CFU/ml resulted in a nearly 5-log reduction after 24 h. The fusion protein, however, was nearly inactive with only a 0.3-log reduction at the same Ply500 concentration and incubation time. We speculated that the decreased activity of the fusion protein could be due to steric hindrance by the larger MBP (44 kDa). To overcome this potential steric limitation, we hypothesized that a short linker between the MBP and the Ply500 could relax this steric constraint.

Our approach took advantage of the presence of a 10 amino acid putative linker between the catalytic and binding domains of Ply500 (Pro-Ala-Ala-Thr-Gln-Asn-Thr-Asn-Thr-Asn)^16^, and we prepared a modified fusion protein containing this 10-mer between the MBP and Ply500 components of the fusion protein (FP_10_). The insertion of a linker improved the cell lytic activity of the fusion protein (Table 1), with 2.5-log reduction observed after 24 h of incubation with 10^5^ CFU/ml *L. innocua*. We then proceeded with affinity immobilization of the FP_10_ onto crosslinked starch nanoparticles. The size of starch nanoparticles was 80 ± 34 nm (as determined by SEM (Supplementary Fig. S5)). A loading of 0.11 ± 0.02 mg protein/mg of starch nanoparticles was obtained for FP_10_. We then evaluated the effect of affinity immobilization on the bactericidal activity of FP_10_ (Table 1); 24% retention in activity (on the basis of the plating assay, e.g., number of listeria cells killed per mg of Ply500) was observed for FP_10_-starch conjugates, with 2.9-log reduction observed after 24 h of incubation with 10^5^ CFU/ml *L. innocua* (Table 1). A control with only MBP attached to starch nanoparticles was inactive. A control consisting of free FP_10_ at 80 μg/ml of Ply500 could not be performed due to rapid formation of visible aggregates in the presence of listeria cells.

To evaluate an actual food material, we tested the efficacy of different Ply500 formulations against exponentially growing *L. innocua* inoculated onto iceberg lettuce leaves. *L. innocua* grew on the lettuce; after 24 h at 25°C the cell density was ca. 10^4^ CFU. Treatment of the contaminated lettuce with free Ply500 (50 μg) or SNP-Ply500 conjugates (200 μg) resulted in complete killing of the contaminating *L. innocua* (Table 2). Less striking results were obtained with the MBP-fusion Ply500, both in the free form and adsorbed to starch. Nonetheless, nearly 1-log unit reduction in CFU was achieved with the FP_10_-starch nanoparticles.

## Discussion

In line with the major requirement of surface-active decontamination methodologies for the food industry, the primary goal of our work was to develop stable and active surface incorporated phage lytic enzyme formulations. We demonstrated the potency of Ply500 by establishing a cell plating assay for *L. innocua* cell killing. Listeriosis treatment includes antibiotics, such as penicillin, ampicillin and amoxicillin^6^,^7^, but their overuse often leads to antibiotic resistance^8^,^9^,^10^. To evaluate whether Ply500 use could lead to gained resistance against *L. innocua* against Ply500 lytic activity, we repeatedly (6 times) exposed *L. innocua* to Ply500 with the expectation that residual viable cells would be more resistant to Ply500 than the original starting stock. However, no change in the log-killing was observed after each round of Ply500 treatment, which suggests that we were not selecting Ply500 resistant strains of *L. innocua*. These results are not surprising. Accelerated evolution of *Bacillus cereus* was performed by Fischetti *et al*^13^ who found no gained resistance of the bacterium upon treatment with a bacillus phage endolysin. While we cannot rule out the ultimate formation of gained resistance of *L. innocua* to Ply500, our treatment indicates that we were not selectively isolating more resistant strains of *L. innocua.*

For surface-based applications of cell-specific endolysins in preventing contact-based bacterial infections, the enzymes must be surface immobilized with long shelf-life and operational reusability. Due to their chimeric structure and requirement of both catalytic and binding domains to be in the proper orientation for lytic activity^17^, the main challenge of this work was to obtain active enzyme after its incorporation onto surfaces. To achieve this goal, we covalently immobilized Ply500 onto FDA approved nanoscale silica particles. Immobilization of Ply500 onto SNPs resulted in enhanced storage stability at 4°C and operational stability at 25°C. This stabilization enabled us to test Ply500-SNP conjugates against *L. innocua* cells not only in buffer but also under exponentially growing conditions on iceberg lettuce leaves. Moreover, we developed an effective listericidal nanocomposite polymer film based on Ply500-SNP conjugates. These films were capable of complete killing of listeria cells on contact and was able to prevent growth of listeria cells at 4°C. This result makes SNP-Ply500-containing pHEMA films potential candidates for applications as anti-listeria coatings of cold storage or food processing equipment, such a thaw and cold rooms, conveyor belts, grinders, commercial kitchens, and packaging materials. Finally, we exploited affinity immobilization of Ply500 on edible crosslinked starch nanoparticles by constructing a MBP-Ply500 fusion protein. These conjugates also exhibited listericidal activity. These Ply500-starch conjugates may find further applications in antimicrobial packaging systems like spraying/coating of antimicrobial enzyme-starch conjugates on food before packaging or incorporation into packaging materials. By extended our approach to other cell lytic enzymes, our findings may encourage broader development of highly efficient bactericidal surfaces for applications in the food industry, healthcare, and other common infrastructures.

## Methods

### Listeria, plasmids and culture conditions

*Listeria innocua* ATCC® 33090™ was used as the cell target in these studies. One shot® Top 10 and One Shot® BL21(DE3) chemically competent *E. coli* cells were obtained from Invitrogen and were used for cloning and overexpression of proteins, respectively. *L. innocua* was grown in Brain Heart Infusion (BHI) medium (BDC, MD, USA) at 37°C with shaking at 220 rpm. From the grown culture, 1 ml was removed and placed in an Eppendorf tube and centrifuged at 10,000 g for 1 min at room temperature to obtain a pellet. The pellet was then washed twice with sterilized PBS to remove excess medium. After washing, the pellet was again resuspended in 1 ml of sterilized PBS. To obtain an approximate measure of cell density in terms of colony forming units (CFU)/ml, the optical density of the microbial suspension was measured at 600 nm (with a conversion factor of 1 absorbance unit corresponding to 10^9^ CFU/ml). The bactericidal efficiency of all the enzyme formulations was determined by using a diluted suspension containing 10^5^ CFU/ml. Unless otherwise indicated, solvents and reagents were obtained from Sigma-Aldrich.

### Cloning procedures

Standard cloning techniques were used for construction of plasmids encoding Ply500 and Ply500-based fusion proteins. The enzymes and kits used for cloning were used according to the manufacturer's instructions.

#### Ply500 plasmid

Ply500 plasmid was obtained by transforming C terminal 6xHis-tagged Ply500 gene in pGS21 vector into *E. coli* (BL21(DE3)) cells.

#### Construction of pMBP-Ply500 (pFP) plasmid

Ply500 gene was amplified by PCR with *Taq* polymerase (Green Master Mix, Promega), using primers Ply500_BamHI_FP (5′CGG CGG ATC CAT GGC ATT AAC AGA GGG ATG) and Ply500_SalI_RP(5′CGC GGT CGA CTT AGT GAT GGT GAT GGT GAT GTT). The amplified gene was then purified by agarose gel extraction using QIAquick Gel extraction kit (Qiagen). The purified Ply500 gene and pHMBP3C vector containing MBP gene were double digested with *Bam* HI and *Sal* I (New England Biolabs) separately for 3h at 37°C. After digestion, the Ply500 gene and MBP plasmid were again purified and the concentration of purified digested gene and plasmid was determined spectrophotometrically (NanoDrop ND-1000). Ply500 gene was ligated into predigested pMBP3C using T4 ligase (New England Biolabs). The ligated plasmid was then transformed into competent One shot® Top 10 cells, which were then plated onto ampicillin-agar plates and incubated at 37°C for 16 h. Plasmids were isolated (Miniprep Kit, Sigma-Aldrich) and sequenced for insertion of Ply500. After confirmation by sequencing, the plasmid containing MBP-Ply500 gene was transformed into competent One Shot® BL21(DE3) *E. coli* cells for overexpression of fusion protein.

#### Construction of pMBP-linker_10_-Ply500 (pFP_10_)

Site directed mutagenesis of plasmid containing MBP-Ply500 was performed (using QuickChange® XL Site-directed Mutagenesis Kit, Agilent) to introduce a 10 amino acid linker between MBP and Ply500. The composition of linker chosen was the same as that of a natural linker that exists between the catalytic and binding domains of Ply500 at positions 154–163; Pro-Ala-Ala-Thr-Gln-Asn-Thr-Asn-Thr-Asn^16^. The primers used were FP: 5′ GGG CCC GGA TCC CCT GCT GCA ACA CAA AAC ACT AAT ACA AAT ATG GCA TTA ACA GAG and RP: 5′ CTC TGT TAA TGC CAT ATT TGT ATT AGT GTT AAG TGT TGC AGC AGG GGA TCC GGG CCC.

### Plating assay of Ply500 based enzyme formulations

Antimicrobial activity of native and immobilized Ply500 was determined by adding an appropriate amount of enzyme formulation (native Ply500 and MBP fusion proteins (20 μg), SNP-Ply500 (60 μg) and FP_10_-starch nanoparticles (80 μg) in 1 ml of PBS (with 0.1% (v/v) Tween 80) containing 10^5^ CFU/ml of *L. innocua.* The presence of surfactant prevents the aggregation of cells and their adhesion to the Eppendorf/petri dish. Following 24 h incubation at 25°C with shaking at 200 rpm, an aliquot was removed and spread onto BHI agar plates. The plates were then incubated for 18 h at 37°C after which CFU's were measured. To evaluate bactericidal effectiveness of Ply500-containing films, a 4.2 × 3.0 × 0.05 cm cutout was subjected to a cell challenge of 10^5^ CFU/ml in 2 ml of PBS buffer containing 0.1% (v/v) Tween 80 in a sterilized Petri dish. CFU determination was performed as described above.

### Modification of silica nanoparticles

Five grams of SNPs (10–20 nm, Sigma-Aldrich) were suspended in 120 ml of 2 M HCl (diluted in 5:1 water-ethanol) and heated to 70°C for 6 h. The acid treated SNPs were centrifuged at 4000 rpm for 30 min and washed with excess deionized water to remove acid, then finally washed with ethanol and dried under vacuum. The product (1 g SNPs) was reacted with 1 ml of a 1:1 mixture of 3-(trimethoxysilyl) propyl methacrylate and 1 ml of 3-(triethoxysilyl) propyl isocyanate in 18 ml of anhydrous cyclohexane under nitrogen^30^. The reaction was carried out overnight at 25°C. The functionalized SNPs (methacrylate-SNP-NCO) were washed several times with cyclohexane by centrifugation (4000 rpm, 30 min), then finally washed with acetone and dried under vacuum. The resulting functionalized SNPs were stored at 4°C and protected from light by wrapping the vial with aluminium foil. For control experiments, SNPs were also functionalized with 3-(triethoxysilyl) propyl isocyanate (SNP-NCO), but without enzyme attachment.

### Immobilization of Ply500 onto SNPs

The Ply500 was covalently immobilized onto SNPs. Briefly, 50 mg of functionalized SNPs (methacrylate-SNP-NCO) were incubated with 5 ml of Ply500 (0.8 mg/mL) in Tris-HCl buffer (pH 8.0, 250 mM NaCl) at 4°C for 10 h. The free amino groups on the enzyme were allowed to react with NCO groups and double bonds on SNPs. After immobilization, SNPs were washed extensively with cold Tris-HCl buffer (pH 8.0, 250 mM NaCl) and 1% Triton × (10 min) at 4°C to remove unbound enzyme. Supernatants and washes were collected to quantify enzyme loading using BCA assay (Pierce, Rockford, IL).

### Preparation of biocatalytic polymeric film

The biocatalytic film was prepared by copolymerizing Ply500-SNP conjugates with 2-hydroxyethyl methacrylate (HEMA) in the presence of polyethylene glycol dimethacrylate (PEGDMA, M_n_ 330) as crosslinker. Methacrylate-SNP-Ply500 (20 mg) conjugates were dispersed in 2.3 mL of Tris-HCl buffer (pH 8.0, 250 mM NaCl) and copolymerized with 200 μL of HEMA in presence of 20 μL of PEGDMA (10% of monomer). Copolymerization was initiated by adding 50 μL of 10% (w/v) of ammonium persulfate (APS) and 10 μL tetramethyl ethylenediamine (TEMED). The copolymerization was carried out for 2 h in gel casting mode (Dimensions, Bio-Rad). The polymeric biocatalytic film thus formed was washed with excess of PBS at 4°C, cut into size of 2.7 × 4.2 × 0.05 cm and further used for anti-listeria activity. The negative control film was also prepared by using methacrylate-SNP-NCO.

### Anti-listeria activity of Ply500 film against adhered *L. innocua* cells at 4°C

Polymer film (2 × 2 cm) was mounted on a glass slide and kept on damp sterile kimwipe tissues in a Petri dish. Polymer film was inoculated with approximately 1000 *L. innocua* cells and incubated at 4°C for 5 h to allow adhesion to occur. Non-adherent bacteria were removed by washing gently with 5 ml of sterile PBS. The film is incubated at 4°C for 24 h after applying 100 μl of BHI media to provide nutrients for the adhering bacteria. After one day incubation, the films were rinsed with sterile PBS and fresh media was added followed by further incubation at 4°C for 24 h. After 2 days, the film was again washed with sterile PBS. To count viable adhered cells, the film was suspended in 1 ml of sterile PBS (with 0.1% (v/v) tween 80) and ultrasonicated for 5 min. The film dispersed to form a homogenous suspension and the plating assay was performed on Palcam listeria-selective agar plates (EMD Chemical In., NJ, USA). A control of listeria cells was ultrasonicated for 5 min. No effect of ultrasonication on listeria cells was observed. Also, due to dispersion of film during ultrasonication, listeria cells were exposed to Ply500-SNP conjugates which were initially buried in the film. So, a control of listeria cells along with same amount of Ply500-SNP present in entire film (2 × 2 cm) was suspended in 1 ml of sterile PBS and was ultrasonicated for 5 min. No significant cell killing was observed by SNP-Ply500 conjugates after ultrasonication for 5 min.

### Affinity immobilization of Ply500 on crosslinked starch nanoparticles

Crosslinked starch nanoparticles (10 mg) were weighed in an Eppendorf tube and 1 ml of 1 mg/ml protein solution (in PBS buffer) was added. The suspension was incubated overnight at 4°C with shaking at 200 rpm. After affinity immobilization of proteins onto the starch nanoparticles, the suspension was centrifuged at 14,000 g for 10 min and supernatant was collected. The starch nanoparticles were then washed five times with cold PBS (1 ml) buffer for 30 min each. The supernatant and washes were assayed for protein concentration and the FP_10_-starch nanoparticle conjugates activity was assessed using the cell wall assay.

### Anti-listeria activity of Ply500 formulations on *L. innocua* inoculated lettuce leaves

Iceberg lettuce was purchased from a local supermarket. The lettuce leaves were cut into size of 4.0 × 3.0 cm and dipped in 70% (v/v) ethanol for 10 min. After washing three times with sterile PBS, lettuce leaves were inoculated with 20 μl of PBS buffer containing ~50 listeria cells. 100 μl of enzyme formulation was applied on inoculated lettuce followed by incubation at RT for 24 h. Lettuce was washed with 1 ml of PBS buffer (containing 0.1% Tween 80) and aliquots of 50, 100 and 200 μl were plated on Palcam listeria selective agar plates (EMD Chemical).

## Author Contributions

J.S.D., R.S.K. and L.S.S. conceived the idea. K.S., N.G., J.S.D. and R.S.K. designed the experiments, analyzed results and wrote the manuscript. K.S. and N.G. performed the experiments. P.D., L.L. and K.K.M. helped in optimization of protein expression and activity. E.E.P. helped in designing molecular biology experiments. All authors discussed the results and commented on the manuscript.

## Supplementary Material

Supplementary Information Supplementary Information

## Figures and Tables

**Table 1:** Anti-listeria activity of different Ply500 formulations against *L. innocua* assessed after incubation with 1 mL of a suspension containing 10^5^ CFU/ml^a^

Ply500 Formulation	Log CFU reduction after 24 h	Retention of native enzyme activity (plating assay) as a percentage relative to native enzyme	Retention of native enzyme activity (cell wall assay) as a percentage relative to native enzyme
Ply500 (20 μg/ml)	4.7 ± 0.05	100%	100%
Ply500-SNP (60 μg/ml)	4.6 ± 0.02	33	n.d.^b^
Ply500 film (500 μg/ml)	1.6 ± 0.12	3.9 ± 0.05	n.d.^b^
FP (20 μg/ml)	0.3 ± 0.02	51 ± 4	4.5 ± 0.5
FP_10 _(20 μg/ml)	2.5 ± 0.30	99 ± 0.5	32 + 4.0
FP_10_-starch nanoparticle (80 μg/ml)	2.9 ± 0.18	24 ± 0.02	n.d.^b^

^a^Listeria cells were grown in BHI media for 7 h. Standard deviations were calculated from triplicate measurements. No significant killing was observed with controls of SNP-NCO, overnight wash of Ply500-SNP, Film (without Ply500), overnight wash of Ply500 film, MBP, starch nanoparticles, and MBP-starch nanoparticles. The concentration (in μg/mL) in column one represents the Ply500 content of the formulation.

^b^not determined.

**Table 2:** Anti-listeria activity of different Ply500 formulations on lettuce inoculated with *L. innocua* cells^a^

Ply500 Formulation	Log CFU after 24 h
Cell control	1.7 ± 0.09
Lettuce + listeria control	4.0 ± 0.05
Ply500 (50 μg)	No cells^b^
Ply500-SNP (200 μg)	No cells^b^
FP_10 _(42 μg)	2.1 ± 0.05
FP_10_-starch nanoparticle (80 μg)	2.8 ± 0.14

^a^Listeria cells were grown in BHI media for 7 h and lettuce was inoculated with 50 *L. innocua* cells. Standard deviations were calculated from triplicate measurements. No significant killing was observed with controls of SNP-NCO, MBP, starch nanoparticles, MBP-starch nanoparticles. The amount (in μg) represents the Ply500 content of the formulation.

^b^No viable *L. innocua* cells were observed in the plating assay.

## References

[b1] Coffey B., Mills S., Coffey A., McAuliffe O. & Ross R. P. Phage and their lysins as biocontrol agents for food safety applications. Annu. Rev. Food Sci. Technol. 1, 449–68 (2010). 22129344 10.1146/annurev.food.102308.124046

[b2] Singh R., Jamieson A. & Cresswell P. GILT is a critical host factor for *Listeria monocytogenes* infection. Nature 455(7217), 1244–1277 (2008). 18815593 10.1038/nature07344 PMC2775488

[b3] McLaughlin H. P., Hill C. & Gahan C. G. M. The impact of iron on *Listeria monocytogenes;* inside and outside the host. Curr. Opin. Biotechnol. 22, 194–199 (2011). 21093251 10.1016/j.copbio.2010.10.005

[b4] Hamon M. A., Ribet D., Stavru F. & Cossart P. Listeriolysin O: the swiss army knife of *Listeria*. Trends Microbiol. 20(8), 360–368 (2012). 22652164 10.1016/j.tim.2012.04.006

[b5] Cox L. J. *et al.* Listeria spp. in food processing, non food and domestic environments. Food Microbiol. 6, 49–61 (1989).

[b6] Janakiraman V. Listeriosis in pregnancy: diagnosis, treatment and prevention. Rev. Obstet. Gynecol. 1(4), 179–185 (2008). 19173022 PMC2621056

[b7] Fernández Guerrero M. L. *et al.* Antimicrobial treatment of invasive non-perinatal human listeriosis and the impact of the underlying disease on prognosis. Clin. Microbiol. Infect. 18(7), 690–695 (2012). 21851486 10.1111/j.1469-0691.2011.03616.x

[b8] Pan Y., Breidt, Jr F. & Kathariou. S. Resistance of Listeria monocytogenes biofilms to sanitizing agents in a simulated food processing environment. Appl. Environ. Microbiol. 72, 7711–7717 (2006). 17012587 10.1128/AEM.01065-06 PMC1694257

[b9] Chen B.-Y., Pyla R., Kim T.-J., Silva J. L. & Jung Y.-S. Antibiotic resistance in Listeria species isolated from catfish fillets and processing environment. Lett. Appl. Microbiol. 50, 626–632 (2010). 20406380 10.1111/j.1472-765X.2010.02843.x

[b10] Pesavento G., Ducci B., Nieri D., Comodo N. & Lo Nostro A. Prevalence and antibiotic susceptibility of *Listeria* spp. isolated from raw meat and retail foods. Food Control 21(5), 708–713 (2010).

[b11] Stasiewicz M. J., Wiedmann M. & Bergholz T. M. The transcriptional response of Listeria monocytogenes during adaptation to growth on lactate and diacetate includes synergistic changes that increase fermentive acetoin production. Appl. Environ. Microbiol. 77(15), 5294–5306 (2011). 21666015 10.1128/AEM.02976-10 PMC3147427

[b12] Yun H. S. *et al.* Susceptibility of Listeria monocytogenes biofilms and planktonic cultures to hydrogen peroxide in food processing environments. Biosci. Biotechnol. Biochem. 76(11), 2008–2013 (2012). 23132560 10.1271/bbb.120238

[b13] Fischetti V. A. Bacteriophage lytic enzymes: novel anti-infectives. Trends Microbiol. 13(10), 491–496 (2005). 16125935 10.1016/j.tim.2005.08.007

[b14] Loessner M. J. Bacteriophage endolysins – current state of research and applications. Curr. Opin. Microbiol. 8, 480–487 (2005). 15979390 10.1016/j.mib.2005.06.002

[b15] Schmelcher M., Donovan D. M. & Loessner M. J. Bacteriophage endolysins as novel antimicrobials. Future Microbiol. 7(10), 1147–1171 (2012). 23030422 10.2217/fmb.12.97 PMC3563964

[b16] Korndörfer I. P. *et al.* Structural analysis of the L-alanoyl-D-glutamate endopeptidase domain of *Listeria* bacteriophage endolysin Ply500 reveals a new member of the LAS peptidase family. Acta. Cryst. D64, 644–650 (2008). 10.1107/S0907444908007890 18560152

[b17] Schmelcher M., Tchang S. & Loessner M. J. Domain shuffling and module engineering of *Listeris* phage endolysins for enhanced lytic activity and binding affinity. Microb. Biotechnol. 4, 651–662 (2011). 21535426 10.1111/j.1751-7915.2011.00263.x PMC3819014

[b18] Gilbreth S. E. *et al.* Relatedness of *Listeria monocytogenes* isolates recovered from selected ready- to- eat foods and listeriosis patients in the United states. App. Environ. Microbiol. 71(12), 8115–8122 (2005). 10.1128/AEM.71.12.8115-8122.2005 PMC1317336 16332793

[b19] Asuri P. *et al.* Increasing protein stability through control of the nanoscale environment. Langmuir 22, 5833–5836 (2006). 16768515 10.1021/la0528450

[b20] Gagner J. E., Lopez M. D., Dordick J. S. & Siegel R. W. Effect of gold nanoparticle morphology on adsorbed protein structure and function. Biomaterials 32 (29), 7241–7252 (2011). 21705074 10.1016/j.biomaterials.2011.05.091

[b21] Shrivastava S., Nuffer J. S., Siegel R. W. & Dordick J. S. Postion-specific chemical modification and quantitative proteomics disclose protein orientation adsorbed on silica nanoparticles. Nano Lett. 12(3), 1583–1587 (2012). 22296027 10.1021/nl2044524

[b22] Resch G., Moreillon P. & Fischetti V. A. A stable phage lysine (Cpl-1) dimer with increased antipneumoccocal activity and decreased plasma clearance. Int. J. Antimicrob. Agents 38, 516–521 (2011). 21982146 10.1016/j.ijantimicag.2011.08.009

[b23] Tang F., Li L. & Chen D. Mesoporous silica nanoparticles: synthesis, biocompatibility and drug delivery. Adv. Mater. 24, 1504–1534 (2012). 22378538 10.1002/adma.201104763

[b24] Asuri P., Bale S. S., Pangule R. C., Shah D. A. Kane R. S. & Dordick J. S. Structure, function, and stability of enzymes covalently attached to single-walled carbon nanotubes. Langmuir. 23, 12318–12321 (2007). 17944500 10.1021/la702091c

[b25] Asuri P., Karajanagi S. S., Vertegel A. A., Dordick J. S. & Kane R. S. Enhanced stability of enzymes adsorbed onto nanoparticles. J. Nanosci. Nanotechnol. 7, 1675–1678 (2007). 17450942 10.1166/jnn.2007.453

[b26] Pangule R. C. *et al.* Biomolecule-nanomaterial interactions: Effect on biolmolecular structure, function and stability. Biological Interactions On Materials Surfaces. 97–114 (2009).

[b27] Godfray H. C. J. *et al.* Food security: The challenge of feeding 9 billion people. Science 327, 812–818 (2010). 20110467 10.1126/science.1185383

[b28] Williams H., Wikström F., Otterbring T., Löfgren M. & Gustafsson A. Reasons for household food waste with special attention to packaging. J. Cleaner Prod. 24, 141–148 (2012).

[b29] Zhao R., Torley P. & Halley P. J. Emerging biodegradable materials: starch- and protein-based bio-nanocomposites. J. Mater. Sci. 43, 3058–3071 (2008).

[b30] Gill I. & Ballesteros A. Degradation of organophosphorous nerve agents by enzyme-polymer nanocomposites: efficient biocatalytic materials for personal protection and large-scale detoxification. Biotechnol. Bioeng. 70, 400–410 (2000). 11005922 10.1002/1097-0290(20001120)70:4<400::aid-bit5>3.0.co;2-2

