# Supplementary material for: Enzyme-Based Listericidal Nanocomposites
Source: Sci Rep. 2013 Apr 2;3:1584. doi: 10.1038/srep01584 (PMC3613805; doi:10.1038/srep01584)
Supplement: Supplementary Information [file srep01584-s1.pdf]

Enzyme-Based Listericidal Nanocomposites

Kusum Solanki[1]*, Navdeep Grover[1]*, Patrick Downs[2], Elena E. Paskaleva[1], Krunal K. Mehta[1], Lillian Lee[1], Linda S. Schadler[2], Ravi S. Kane[1#], Jonathan S. Dordick[1#]

[1]Department of Chemical and Biological Engineering, [2]Department of Materials Science and Engineering
Center for Biotechnology & Interdisciplinary Studies, and Rensselaer Nanotechnology Center
Rensselaer Polytechnic Institute, Troy, NY 12180, USA

*****Equal contribution

# Corresponding authors:

Jonathan S. Dordick
Departments of Chemical and Biological Engineering, Materials Science and Engineering, Biomedical Engineering, and Biology
Rensselaer Polytechnic Institute
2213 Center for Biotechnology & Interdisciplinary Studies
110 8th Street
Troy, NY 12180, USA
Ph: + (1) 518-276-2899
Fax: + (1) 518-276-2207
E-mail: dordick@rpi.edu

Ravi S. Kane
Department of Chemical and Biological Engineering
Rensselaer Polytechnic Institute
4105 Center for Biotechnology & Interdisciplinary Studies
110 8th Street
Troy, NY 12180, USA
Ph: + (1) 518-276-2536
Fax: + (1) 518-276-4030
E-mail: kaner@rpi.edu

Materials and Methods

Overexpression and purification of Ply500 and MBP-Ply500 fusion proteins

A colony of *E. coli* (BL21(DE3)) cells containing C-terminal 6xHis-tagged Ply500 gene in pGS21 or MBP-Ply500 gene in pMBP3C was inoculated into 50 ml of Luria-Bertani (LB) medium (Sigma Aldrich) supplemented with 0.1 mg/ml of ampicillin (Gold Biotechnology). The culture was grown overnight at 37°C and 220 rpm, after which 10 ml was inoculated into 250 ml of LB media also supplemented with 0.1 mg/ml Ampicillin. The resulting culture was grown at 37°C and 220 rpm until the optical density at 600 nm reached 0.6. Ply500 expression was then induced with 1 mM isopropyl β-D-1-thiogalactopyranoside (IPTG, Gold Biotechnology) followed by incubation at 37°C for 3 h. In the case of MBP-Ply500 fusions, the culture was induced with 0.5 mM IPTG followed by incubation at 25°C for 10 h. Cells were harvested by centrifugation at 4000g for 30 min at 4°C. The supernatant was discarded and the pellet was resuspended in 15 ml of native purification buffer (NPB, Tris-HCl buffer, pH 8.0) containing 0.1 mg/ml lysozyme and 10 µg/ml of DNase I. The suspension was sonicated for 10 min with 60% amplitude and cycles of 10 s. The cell debris was removed by centrifugation at 4000g for 40 min at 4°C. The supernatant was then added to a Ni-NTA resin (4 ml) in a 20 ml column. After washing with NPB containing 20 mM imidazole, the Ply500-MBP fusion was eluted with NPB containing 250 mM imidazole. The eluted protein was then concentrated in 50 mM Tris-HCl buffer containing 250 mM NaCl using 3 kDa Amicon-Ultra Centrifugal Device (Millipore). Finally, purified and concentrated Ply500-MBP fusion was filter sterilized using 0.22-µm syringe filter (Millipore) and was quantified for protein using the BCA Protein Kit Assay (Thermo Fischer Scientific). The sterilized protein (< 1 mg/ml) was then stored at 4°C until use.

Size exclusion FPLC analysis

The existence of Ply500 as monomer, dimer or oligomer was studied by size exclusion chromatography using fast protein liquid chromatography (AKTA Purifier, GE Healthcare). Protein samples (2 ml) with concentration of 0.5 and 1 mg were loaded into Superdex 200 column (GE Healthcare) and then eluted with 50 mM Tris-HCl buffer (pH 8.0) containing 250 mM NaCl with a flow rate of 0.5 ml/min. Column calibration was performed by running a mixture of three proteins: Bovine serum albumin (65.4 KDa), Deoxyribonuclease I (31.3 KDa) and Ribonuclease A (15.6 KDa).

Spectrophotometric assay of Ply500 using isolated listeria cell wall

The lytic activity of Ply500 and fusion proteins were determined by a photometric lysis assay as described by Schmelcher et al.[1]. Briefly, cell wall material was isolated from *L. innocua* following the literature protocol[2]. Cell lysis was performed in a total volume of 1 ml with cell wall diluted in 50 mM Tris-HCl buffer (pH 8.0, 250 mM NaCl) to an initial OD_{600} between 0.30 to 0.35. After addition of enzyme (concentration ranging from 0.5-2.0 µg/ml), the decrease in OD_{600} was measured for 6-7 min. All experiments were performed in triplicate and the average slope was used to calculate activity units. One unit of activity was defined as the amount of enzyme required to decrease the OD_{600} in suspension by 0.01/min[1].

Screening of resistance of *L. innocua* against Ply500

Ply500 (10 µg) was incubated with *L. innocua* (10^5 CFU/ml) in 1 ml of sterile PBS (with 0.1 % (v/v) Tween 80) for 24 h at 25°C. An aliquot (100 µl) was removed and spread onto Palcam Listeria selective agar plates (EMD chemical In., NJ, USA). The plates were then incubated for 18 h at 37°C. One colony is picked up and grown again in BHI media for 7 h. The listeria cells

thus grown were again incubated with Ply500 as described above. This sequence was repeated six times.

Determination of methacrylate groups on SNPs

The presence of methacrylate groups on functionalized SNPs was determined qualitatively by X-ray photoelectron spectroscopy (PHI 5000 Versaprobe). The double bond of methacrylate group was reacted with bromine (Sigma) in anhydrous THF. Briefly, 50 mg of methacrylate-SNP-NCO were reacted overnight with 2 ml of bromine in 3 ml of THF under nitrogen at room temperature. After reaction, SNPs were washed with excess THF by centrifugation (10000 rpm, 20 min) to remove the unreacted bromine. The washed SNPs were then suspended in acetone and drop cast on silicon wafer. The acetone was allowed to evaporate and the samples were immediately analyzed. Unfunctionalized SNPs and SNP-NCO were used as negative controls. The presence of reacted bromine i.e. C-Br bond was identified by XPS.

Determination of isocyanate and methacrylate groups on SNPs

The total number of isocyanate and methacrylate groups on SNPs were quantified by titration with benzylamine[3,4]. Briefly, benzylamine (0.35 ml, 3.2 mmol) was added to the methacrylate-SNP–NCO/ SNP-NCO (1 g) dispersed in 20 ml of THF. The reaction was performed in the presence of a trace amount of dibutyltin dilaurate as catalyst at 60˚C for 24 h. The amount of unreacted benzylamine was determined by titrating with 0.01 M HCl using bromophenol as an indicator (end point blue to greenish yellow). Therefore, the number of moles of isocyanate groups on methacrylate-SNP–NCO/ SNP-NCO is equal to the difference of moles of starting benzylamine and unreacted benzylamine. Unfunctionalized SNPs also were reacted with benzyl amine as negative controls and titrated with HCl.

Leaching studies

Potential leaching of Ply500 from the film was assessed by washing Ply500-SNPs based polymer film in 2 ml of PBS buffer (containing 0.1% (v/v) Tween 80) for 24 h. The supernatant was then assayed for Ply500 activity using the aforementioned plating assay. The leaching of enzyme from polymeric film was also studied by using GFP as a model enzyme. GFP was covalently immobilized onto SNPs and GFP-SNP conjugates were incorporated into the polymer film via the aforementioned methodology. The film was washed for 24 h in 2 ml of PBS containing 0.1% (v/v) Tween 80. The overnight film wash was assessed for fluorescence (Ex: 490 nm, Em: 540 nm).

Storage stability of Ply500-SNP conjugates

The stability of Ply500-SNP conjugates in Phosphate Buffered Saline (PBS) was tested at 4°C and 25°C. Freshly prepared Ply500-SNPs were stored in PBS for 45 and 30 days at 4°C and 25°C respectively. Activity measurements using plating assay were done periodically to evaluate enzyme stability. Relative log killing (%) was calculated as

Relative log killing (%) = (log reduction obtained after storage for n days)x100/(log reduction obtained at 0 day)

Preparation of crosslinked starch nanoparticles

The crosslinked starch nanoparticles were prepared as reported in Ma *et al.*[5]. Briefly, corn starch (1g) was suspended in 20 ml of distilled water and heated at 90°C with constant stirring until it dissolved completely. Ethanol (20 ml) was then added dropwise with constant stirring. The nanoparticles thus formed were cooled to room temperature followed by further dropwise

addition of 20 ml of ethanol with constant stirring. The suspensions were then centrifuged at 8000 rpm for 20 min. After two washes with ethanol, the starch nanoparticles were dried at 37°C to remove all residual ethanol.

Starch nanoparticles (0.5 g) were suspended in 3 ml of citric acid solution (0.3 g/ml in ethanol) in a glass Petri dish. The suspension was kept at 25ºC for 12 h so that the nanoparticles absorb the citric acid. The Petri dish was then heated at 50°C for 6 h to remove ethanol. The starch nanoparticles were then heated at 130°C for 2 h. At this high temperature, the carboxyl groups of citric acid react with hydroxyl groups of starch to form ester bonds resulting in cross linking of starch nanoparticles. The dry nanoparticles were then washed three times with ethanol to remove water, dried at room temperature and grounded in a mortar and pestle. The cross-linked starch nanoparticles were then characterized for their size using SEM (Supplementary Fig. S5).

Characterization of nanocomposite films and cross-linked starch nanoparticles by Scanning Electron Microscopy (SEM)

Distribution of Ply500 immobilized SNPs in the film, film topography and size distribution of starch nanoparticles were studied via SEM using a JEOL-JSM 840 Scanning Electron Microscope with field emission gun operated at 10.0 kV. Films containing Ply500-SNPs fused along with the monomer and starch nanoparticles were deposited on a silica wafer and dried under vacuum overnight and mounted on an SEM sample holder using a double sided carbon tape and imaged. Particle size of crosslinked starch nanoparticles was obtained SEM image using imageJ. Average size was obtained by measuring the size of at least 50 nanoparticles each from SEM images of three different preparations.

Dynamic Light Scattering (DLS)

DLS measurements were performed using a DynaPro-Titan Batch DLS instrument (Wyatt Technology Corp.) and the DYNAMICS software was used to resolve the acquisitions into a well-defined distribution of hydrodynamic radii. The samples were run in PBS buffer (containing 0.1 % (v/v) tween 80). DLS values for each sample were averaged over 10 runs of 10 measurements per run at 25°C.

References

1. Schmelcher, M., Tchang S. & Loessner, M.J. Domain shuffling and module engineering of *Listeris* phage endolysins for enhanced lytic activity and binding affinity. *Microb. Biotechnol*. **4,** 651-662 (2011).

2. Schmelcher , M., Waldherr , F. & Loessner, M.J. Listeria bacteriophage peptidoglycan hydrolases feature high thermoresistance and reveal increased activity after divalent metal cation substitution. *Appl. Microbiol. Biotechnol.* **93,** 633-643 (2012).

3. Zhao, W., Pacard, E., Chaix-Bauvais, C., Pichot, C. & Brook, M.A. Covalent assembly of silica nanoparticle aggregate for oligonucleotide synthesis. *Colloids Surface A* **339,** 26-34 (2009).

4. Vadala, M.L. *et al*. Cobalt-silica magnetic nanoparticles with functional surfaces. *J. Magnetism Magnetic. Mater*. **293,** 162-170 (2005).

5. Ma, X., Jian, R., Chang, P.R. & Yu, J. Fabrication and characterization of citric acid-modified starch nanoparticles/plasticized-starch composites. *Biomacromolecules* **9,** 3314-3320 (2008).

6. Guanfan, X., Xiaoguang, S., Xuepeng, Q., Jinalan, Z. & Guodong, Z. XPS studies on surface modified poly[1-(trimethylsilyl)-1-propyne] membranes II surface modification by bromine vapor. Chinese J. Ppl. Sci., **12 (2),** 180-184 (1994).

Supplementary Table S1: Size of different preparations of SNPs determined using DLS.

SNPs	Size (diameter) (nm)
Unmodified SNPs	91 ± 31
Acid treated SNPs	46 ± 21
NCO and methacrylate functionalized SNPs	65 ± 18
Ply500-SNPs	81 ± 29

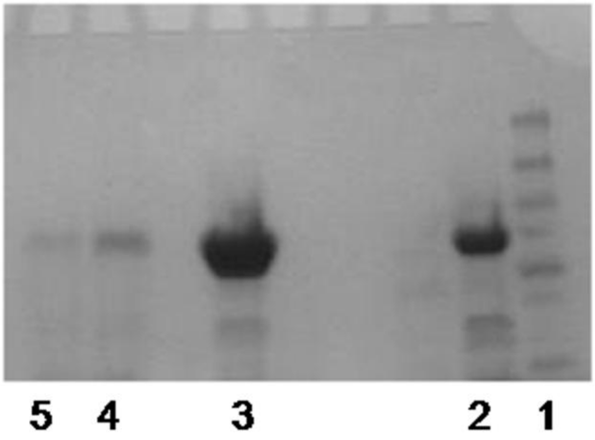

Supplementary Figure S1: Coomassie-stained sodium dodecylsulphate polyacrylamide (SDS-PAGE) gel of Ply500 elute fractions from size exclusion chromatography. Lane1 – molecular weight marker; 2 – purified Ply500; 3- peak corresponding to monomer of Ply500 obtained by gel filtration on sephadex G-100; 4- peak corresponding to dimer of Ply500 obtained by gel filtration on sephadex G-100; 5- peak corresponding to aggregates of Ply500 obtained by gel filtration on sephadex G-100. In SDS-PAGE gel, the bands for all these fractions were observed at ~33KDa indicating that monomer, dimer and aggregate peaks were of Ply500, which disaggregated into monomer only when the samples were boiled in presence of SDS and β-mercaptoethanol for SDS-PAGE gel analysis.

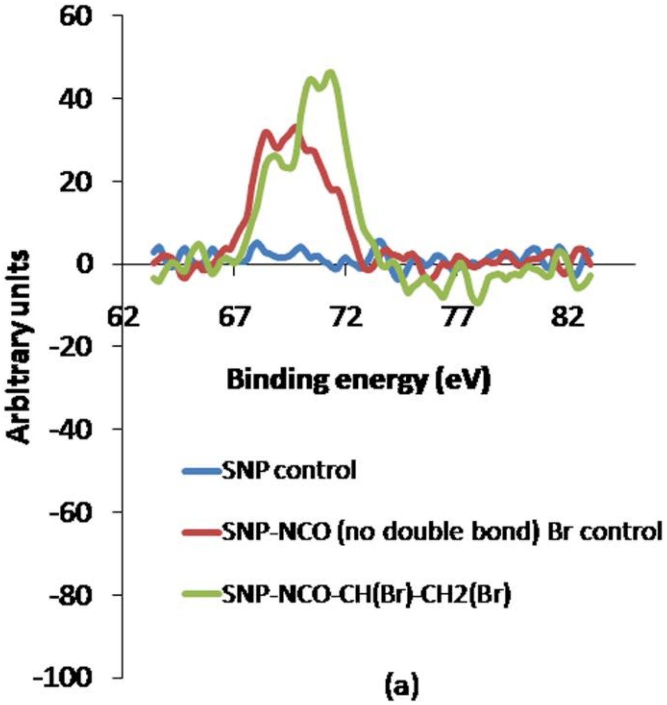

(a)

SNPs	Atomic percentage of bromine
SNP control	0
SNP-NCO-(no double bond)-bromine control	1.6
SNP-NCO-CH(Br)-CH_2(Br)	3.5

(b)

Supplementary Figure S2: X-ray Photoelectron spectroscopy analysis of SNPs to characterize the presence of methacrylate groups (double bonds) **a)** Comparison of Br 3d spectra of functionalized SNPs **b)** Atomic percentages of Bromine present in SNP samples calculated from spectra in figure (**a**). Br attached on silicon has binding energy of 69 eV whereas Br attached to C (i.e C-Br) has binding energy of 71 eV[6]. Following treatment with Br_2, SNP-NCO control showed a peak at 69 eV, which is due to Br-Si bond whereas (CH_2=CH)-SNP-NCO, showed peak at 71 eV which is due to C-Br bond. SNP control (which was not treated with Bromine) did not show any peak of Br.

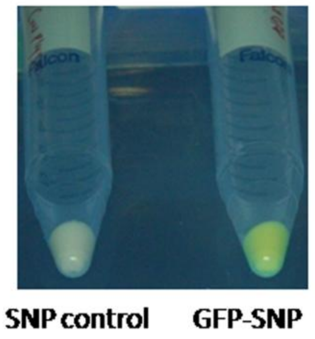

(a)

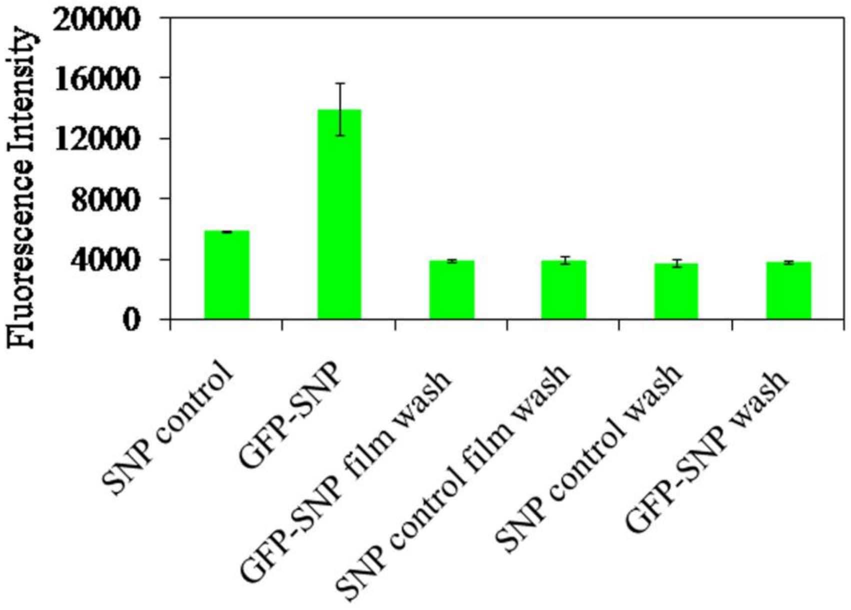

(b)

Supplementary Figure S3: (a) Covalent immobilization of GFP on SNPs (b) Fluoroscence measurements for GFP leaching in overnight washes of SNP-GFP conjugates and polymer film made by incorporating SNP-GFP conjugates by co-polymerization with HEMA.

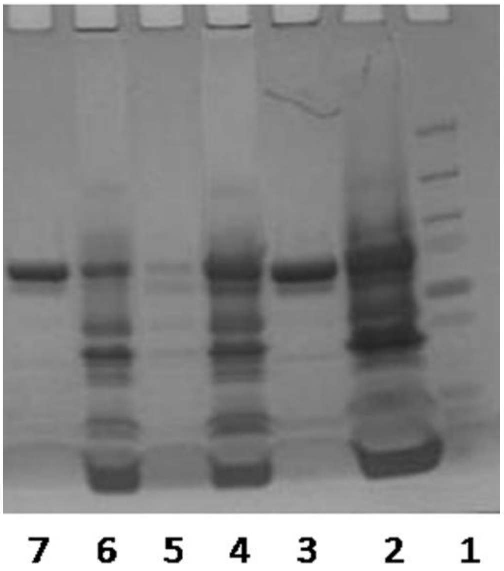

Supplementary Figure S4: Coomassie-stained sodium dodecylsulphate polyacrylamide (SDS-PAGE) gel of Ni-NTA purified fusion proteins MBP-Ply500 and MBP-linker$_{10}$-Ply500. Lane 1: Molecular weight marker; Lane 2: Total cell lysate of MBP-Ply500 fusion protein (without linker); Lane 3: Purified MBP-Ply500 fusion protein (without linker); Lane 4: Total cell lysate of MBP-linker-Ply500 fusion protein (with linker); Lane 5: Column wash of MBP-linker-Ply500 fusion protein (with linker); Lane 6: Flow through of MBP-linker-Ply500 fusion protein (with linker); Lane 7 : Purified MBP-linker-Ply500 fusion protein (with linker).

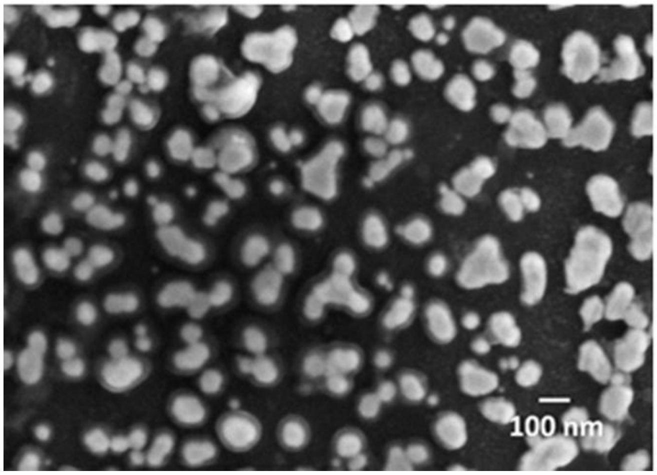

Supplementary Figure S5: SEM picture of crosslinked starch nanoparticles.
